# Digital Peer Support Mental Health Interventions for People With a Lived Experience of a Serious Mental Illness: Systematic Review

**DOI:** 10.2196/16460

**Published:** 2020-04-03

**Authors:** Karen L Fortuna, John A Naslund, Jessica M LaCroix, Cynthia L Bianco, Jessica M Brooks, Yaara Zisman-Ilani, Anjana Muralidharan, Patricia Deegan

**Affiliations:** 1 Dartmouth College Lebanon, NH United States; 2 Department of Global Health and Social Medicine Harvard Medical School Harvard University Boston, MA United States; 3 Department of Medical & Clinical Psychology Uniformed Services University of the Health Sciences Rockville, MD United States; 4 The Giesel School of Medicine Dartmouth College Concord, NH United States; 5 Geriatric Research, Education and Clinical Center James J Peters VA Medical Center New York, NY United States; 6 Department of Social and Behavioral Sciences College of Public Health Temple University Philadelphia, PA United States; 7 Pat Deegan and Associates Boston, MA United States

**Keywords:** peer support, digital mental health, recovery

## Abstract

**Background:**

Peer support is recognized globally as an essential recovery service for people with mental health conditions. With the influx of digital mental health services changing the way mental health care is delivered, peer supporters are increasingly using technology to deliver peer support. In light of these technological advances, there is a need to review and synthesize the emergent evidence for peer-supported digital health interventions for adults with mental health conditions.

**Objective:**

The aim of this study was to identify and review the evidence of digital peer support interventions for people with a lived experience of a serious mental illness.

**Methods:**

This systematic review was conducted using Preferred Reporting Items for Systematic Reviews and Meta-Analyses (PRISMA) procedures. The PubMed, Embase, Web of Science, Cochrane Central, CINAHL, and PsycINFO databases were searched for peer-reviewed articles published between 1946 and December 2018 that examined digital peer support interventions for people with a lived experience of a serious mental illness. Additional articles were found by searching the reference lists from the 27 articles that met the inclusion criteria and a Google Scholar search in June 2019. Participants, interventions, comparisons, outcomes, and study design (PICOS) criteria were used to assess study eligibility. Two authors independently screened titles and abstracts, and reviewed all full-text articles meeting the inclusion criteria. Discrepancies were discussed and resolved. All included studies were assessed for methodological quality using the Methodological Quality Rating Scale.

**Results:**

A total of 30 studies (11 randomized controlled trials, 2 quasiexperimental, 15 pre-post designs, and 2 qualitative studies) were included that reported on 24 interventions. Most of the studies demonstrated feasibility, acceptability, and preliminary effectiveness of peer-to-peer networks, peer-delivered interventions supported with technology, and use of asynchronous and synchronous technologies.

**Conclusions:**

Digital peer support interventions appear to be feasible and acceptable, with strong potential for clinical effectiveness. However, the field is in the early stages of development and requires well-powered efficacy and clinical effectiveness trials.

**Trial Registration:**

PROSPERO CRD42020139037; https://www.crd.york.ac.uk/PROSPERO/display_record.php?RecordID= 139037

## Introduction

### Background

Peer support is recognized globally as an essential recovery service for people with mental health conditions [1]. Peer support services are recovery and wellness support services provided by an individual with a lived experience of recovery from a mental health condition [2]. Peer support is broadly defined as “giving and receiving help founded on key principles of respect, shared responsibility, and mutual agreement of what is helpful” [3], and such services have proven to be instrumental in augmenting traditional mental health treatment [3], thereby providing effective recovery services to people with mental health conditions [4,5]. In particular, peer support services have contributed to increases in patient engagement, positive medical outcomes, patient activation, and greater use of self-management techniques [4,5]. In the largest randomized controlled trial of a peer-led, self-management intervention conducted to date, the researchers found improved physical health and mental health-related quality of life among individuals with serious mental illness and comorbid medical conditions [6]. With the influx of digital mental health services changing the way mental health care is delivered, peer supporters are increasingly using technology to deliver peer support [7].

### Digital Peer Support Mental Health Interventions

Traditionally, peer support has been provided as an in-person intervention in multiple service settings such as inpatient and outpatient psychiatric units [3]. More recently, peer support is increasingly being offered through digital technologies, known as digital peer support. Digital peer support is defined as live or automated peer support services delivered through technology media such as peer-to-peer networks on social media, peer-delivered interventions supported by smartphone apps, and asynchronous and synchronous technologies; asynchronous technology facilitates communication between peer support specialists and service users without the need for communication to happen in real time [8]. Through these mobile and online technologies, adoption of digital peer support is expanding the reach of peer support services [8], increasing the impact of peer support without additional in-person sessions [9], and engaging service users in digital mental health [10]. Peers are also co-producing empirically supported digital peer support services [11]. For example, peers working in equal partnership with academic researchers developed [11] and tested a smartphone-based medical and psychiatric self-management intervention for people with mental health conditions, which contributed to statistically significant improvements in psychiatric self-management [9,12]. In addition, improvements were observed in self-efficacy for managing chronic health conditions, hope, quality of life, medical self-management skills, and empowerment [9,12]. Given these advances, there is a need to review and synthesize the emerging evidence for digital peer support interventions for adults with mental health conditions.

Our objectives were (1) to expand on prior reviews that focused on peer support services that did *not* include technology [4,5] or that focused on peer support using technology but *only for* people with psychosis [13] and (2) to conduct a systematic literature review to assess the feasibility, acceptability, and potential effectiveness of digital peer support interventions for adults with serious mental illnesses. We examined the effect of interventions on both biomedical and psychosocial outcomes. In addition, we examined the extent to which researchers engaged service users in the development of the identified digital peer support interventions.

## Methods

### Search Strategy

We followed the PRISMA (Preferred Reporting Items for Systematic Reviews and Meta-Analyses) procedures [14]. Our search strategy protocol was published to the PROSPERO International prospective register of systematic reviews (Registration number: CRD42020139037). To identify early peer-reviewed articles reporting on digital peer support interventions, we included the following available high-quality electronic reference databases beginning in 1946 until December 2018: PubMed, Embase, Web of Science, Cochrane Central, CINAHL (Cumulative Index to Nursing and Allied Health), and PsycINFO. Each search term was entered as a keyword and assigned the corresponding Medical Subject Heading term (see Multimedia Appendix 1 for the full list of search terms). To identify articles not included in our original search, we reviewed the reference lists of published studies that met the inclusion criteria along with prior systematic reviews, and in June 2019, we searched Google Scholar using different combinations of the search terms.

### Study Selection Criteria

Studies were evaluated by the first two authors (KF and JN) who independently screened titles and abstracts. We piloted our title and abstract review protocol on 15 references to ensure 100% concordance/agreement between reviewers before reviewing the entire set of titles and abstracts. These authors independently reviewed all full-text articles meeting the inclusion criteria. Any discrepancies were discussed and resolved. According to the PRISMA guidelines [14], we used the participants, interventions, comparisons, outcomes, and study design (PICOS) criteria [15] to assess study eligibility:

- Participants: Individuals aged≥18 years with either a diagnosis of schizophrenia spectrum disorder (schizophrenia or schizoaffective disorder) or bipolar disorder.
- Intervention: Digital peer support interventions, including peer-delivered interventions, peer augmented interventions, and peer-to-peer social media interventions.
- Comparisons: Studies did not need to have a comparison condition. Interventions could have been delivered at any location such as participants’ homes, primary care setting, federally qualified health centers, outpatient facilities, inpatient facilities, community mental health centers, community settings, or could have been delivered via remote or mobile technology.
- Outcomes: The primary outcomes of interest included those related to feasibility, acceptability, and effectiveness (ie, biomedical and psychosocial outcomes).
- Study design: We included randomized controlled trials, pre-post designs with an experimental or a quasi-experimental comparison condition, qualitative studies, and secondary data analyses if outcomes were relevant to the feasibility, acceptability, and effectiveness of digital peer support interventions. Research protocols, letters to the editor, review articles, pharmacological studies, theoretical articles, and articles that were not peer-reviewed were excluded from this systematic review.

### Data Extraction

Relevant data from included studies were extracted in duplicate by two reviewers (KF and JN) using a standardized data collection tool. Prior to data extraction, the two reviewers piloted the data collection tool on five included articles to identify and reconcile inconsistent findings or unintended omission of data. A third reviewer (JB) approved the final set of data, decided on any of the remaining data discrepancies, and extracted study characteristics. Extracted study characteristics included study design, sample size and attrition, participant sociodemographic and clinical characteristics, length of study, description of comparison or control group, physical location of intervention (eg, community mental health centers, Veterans Affairs), a description of the intervention, and outcomes.

In addition to the characteristics listed above, we extracted information regarding the extent to which service users were engaged and participated in the development of intervention components. As no benchmark of participant engagement has been consistently defined in the scientific literature, participation rates were divided to present the spread of data. Participation rates were categorized as high engagement (75% or more engaged throughout the intervention), medium engagement (74% to 50% engage throughout the intervention), and low engagement (49% or less engage throughout the intervention). In the event that percentages were not reported or could not be determined, the authors classified studies based on the information provided (eg, study reported statistically significant levels of engagement).

Studies were further categorized by service delivery type, including peer-to-peer networks, peer-delivered interventions supported by technology, and synchronous and asynchronous technologies.

### Methodological Quality Assessment

All included studies were assessed for methodological quality using the Methodological Quality Rating Scale (MQRS) [16], which assesses 12 methodological attributes of quality and has been used in other systematic reviews [17-19]. Cumulative scores range from 0 (poor quality) to 17 (high quality); studies that receive a cumulative score of at least 14 are considered to be high-quality studies [16]. Two authors (JB and CB) independently completed the MQRS for studies that met the inclusion criteria. Discrepancies in MQRS ratings were addressed and resolved by the first two authors (KF and JN).

## Results

### Included Studies

**Figure 1:**
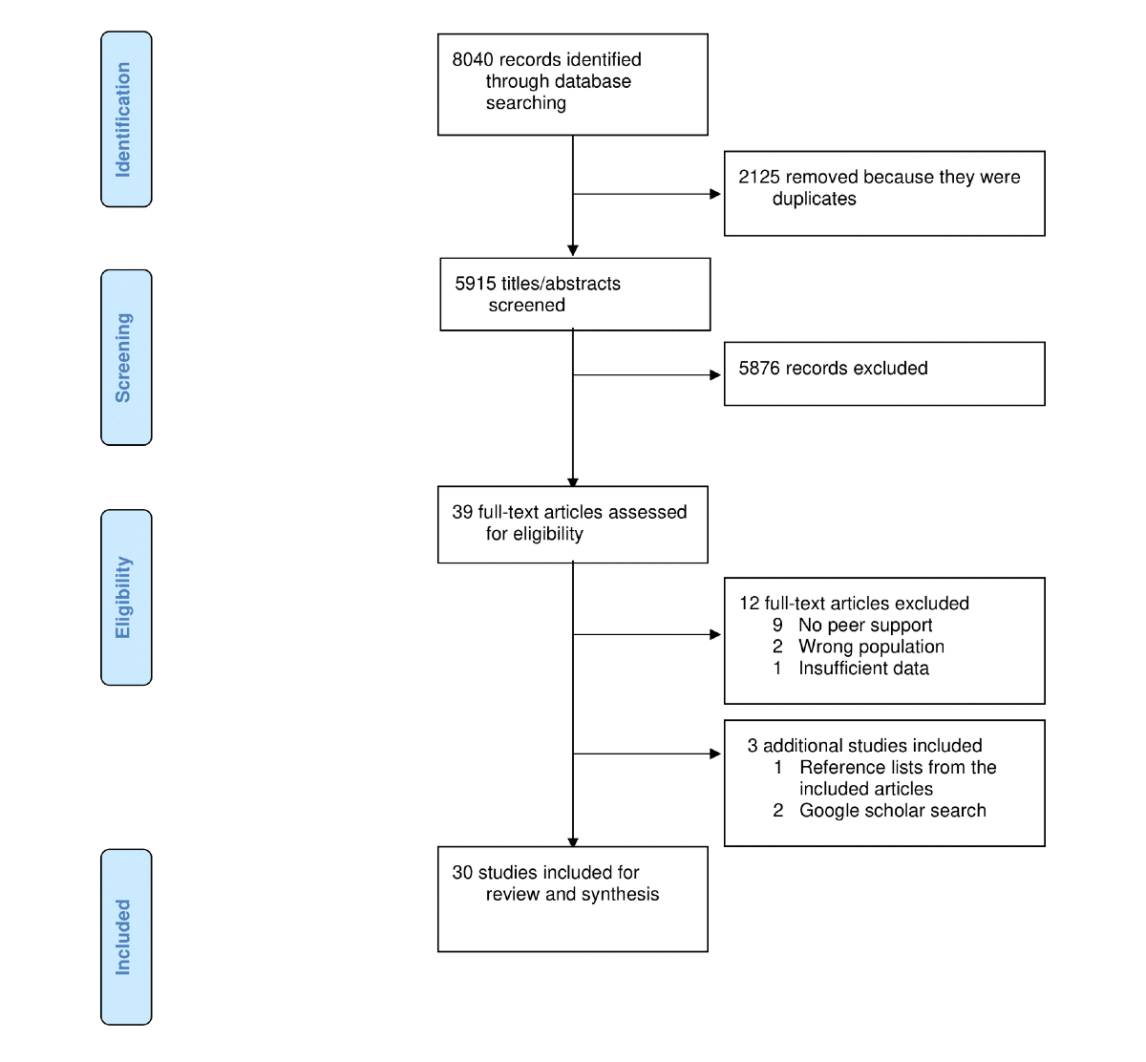
PRISMA (Preferred Reporting Items for Systematic Reviews and Meta-Analyses) flow diagram of studies included in the review.

The search strategy identified 8030 articles, including 2125 duplicates. Of the total 5915 titles and abstracts reviewed, 5876 did not meet the inclusion criteria. The full texts of the remaining 39 articles were assessed, and 12 did not meet the inclusion criteria. None of the non–English language articles met the inclusion criteria. Additional articles were found by searching the reference lists of the 27 articles that met the inclusion criteria and conducting a Google Scholar search in June 2019, resulting in an additional 3 included articles. Overall, 30 articles describing 24 interventions met the inclusion criteria and were included in this review (see Figure 1).

As indicated above, included interventions were categorized by the service delivery type by one author (KF). Overall, 14 studies examined peer-to-peer networks, 11 studies examined peer-delivered interventions supported with technology, 2 studies examined peer-supported interventions using synchronous technology, and 3 studies examined peer-supported interventions using asynchronous technology.

### Peer-to-Peer Networks

Informal peer support, also known as a “peer-to-peer network” or commonly referred to in the medical community as a “patient-facilitated network,” is defined as support given between people with similar life experiences [8]. For example, informal peer support can naturally occur among people in a one-on-one discussion, in a group, or digitally. Informal peer support does not require education or training; rather, people with similar lived experiences define these interactions (see Multimedia Appendix 2). Below, we describe identified studies that were categorized as either stand-alone peer-to-peer networks or peer-to-peer networks combined with evidence-based practices.

#### Peer-to-Peer Networks

We found one study, a randomized controlled trial, that implemented a peer-to-peer network using a peer support listserv (unmoderated, unstructured, anonymous) and a peer support bulletin board [20]. Although this study was feasible and acceptable, the researchers found no differences between the experimental and control groups on any of the outcomes of interest, including quality of life, empowerment, social support, or psychiatric symptoms.

#### Peer-to-Peer Networks Combined With Evidence-Based Practices

We found 13 studies that implemented a peer-to-peer network in combination with evidence-based practices [21-31]. These studies included pre-post studies [25-30] and not fully powered randomized controlled trials [21,31] that were designed to address self-management [31], social cognition training [28], weight management [25,26,29,30], motivational enhancement [24], psychoeducation [22,23], or parenting skills training [21,32]. Peer support was facilitated through Facebook [24-26], Google Docs [33], internet-based bulletin boards [20,27], listserv [20,32], or smartphone apps [24,28]. Three studies combined peer-to-peer networks with fitness trackers to promote self-monitoring of physical activity and exercise [25,30].

Overall, these studies appeared feasible. However, attrition rates varied widely. Among the studies that reported attrition, the attrition rates of in-person studies ranged from 0% to 78% [24-28]. Among studies that reported attrition in the technology portion of the study, the attrition rates remained relatively constant: one study using Facebook reported 24% attrition [30] and studies using internet-based bulletin boards reported 0%-5% attrition [22,27].

Participants in studies of interventions consisting of peer-to-peer networks combined with evidence-based practice interventions reported statistically significant improvements in psychiatric symptoms (ie, fewer positive symptoms [22,23] and fewer depression symptoms [24,27]), self-management and biometric outcomes (ie, self-efficacy, weight loss, decreased body mass index [25], clinically significant improvements in cardiovascular fitness [25,26]), person-reported outcomes (ie, improved patient satisfaction [24,26]), service utilization (ie, decreased hospital admissions and hospital length of stay [29]), knowledge (ie, significant increase in knowledge about schizophrenia [22,23]), parenting (ie, improved skills and satisfaction [21,32]), and psychosocial processes (ie, reduced maladaptive social cognitions [28] and improved motivation [24]).

### Peer-Delivered Interventions Supported With Technology

#### Overview

We found 11 studies that implemented peer-delivered interventions supported with technology [9,34-43]. These studies included 1 qualitative study [40], 5 pre-post studies [9,34,36,42,43], 3 quasiexperimental studies [35,38,39], and 2 randomized controlled trials [37,41] that aimed to address integrated medical and psychiatric self-management [9], shared decision making [34,43], cognitive enhancement therapy [41], physical well-being [38,39], and weight management [42]. Peer-delivered services were delivered through smartphone apps [38,43], in-person and augmented by a smartphone app [9,40], in-person and augmented by text messaging and a fitness tracker [42], or via a web-based platform with a peer [34,41] (see Multimedia Appendix 3).

Overall, these studies seem to also be feasible, with the exception of one study [43], in which the mode of delivery (ie, smartphone app) was deemed not feasible. However, attrition rates varied greatly, ranging from 0% to 77% [9,34,35,38,39,41,42].

There was a wide variety of reported outcomes in the peer-delivered interventions supported with technology. Below, we present the statistically significant outcomes, qualitative outcomes, and null results.

#### Statistically Significant Outcomes

Participants who completed the peer-delivered interventions supported with technology experienced statistically significant benefits in shared decision–making reports [34,37], health care utilization (ie, improvement in engagement in mental health outpatient services [35] and provider perceptions of consumer involvement [37]), self-management (ie, improved medication adherence [35] and psychiatric self-management [9]), person-reported outcomes (ie, improvement in recovery, self-reported psychiatric symptoms [36], lower medication side effects [37]), and patient experience (ie, better relationship and communication between users and doctors [37])*.* In addition, in one study, the presence of a peer support specialist was associated with better cognitive performance among participants with a lived experience of a serious mental illness completing computerized neurocognitive remediation training sessions [41].

#### Qualitative Outcomes

Service users and providers reported finding the app in one study useful for supporting people in recovery via its ability to provide an overview of the intervention and set a treatment agenda, while promoting a connection with peer support specialists [40]. Two studies found technological obstacles to the use of technology defined as frustration with technical malfunctions in the app [40,43].

#### Null Results

Some studies found modest improvements (not statistically significant) in hope, empowerment, social support, quality of life [9], self-reported physical health status [38], and weight loss [42]. Some studies did not find changes in outcomes as related to walking, self-reported global health quality, mental health quality, health control, mental health control, stages of change for exercise [38], self-reported treatment involvement, hope, self-reported patient activation and autonomy preferences [36], patient activation, patient satisfaction, psychiatric distress, global assessment of functioning, drug-induced extrapyramidal symptoms, medication adherence, and quality of life [37]. One study reported low levels of patient satisfaction with an app [39].

### Asynchronous and Synchronous Technologies

#### Synchronous Technologies

As shown in Multimedia Appendix 4, we found 2 articles that reported on a fully powered randomized controlled trial using synchronous technologies [44,45]. In these studies, peer support was facilitated through the telephone [45] combined with internet-based modules accessible at Veteran Affairs clinic kiosks. Overall, the intervention appeared feasible; however, only 86/276 (31.2%) of enrolled participants completed the intervention [44,45]. Participants who attended at least one session of the intervention, whose weight was in the obese range, and who completed synchronous technology components reported statistically significant benefits [45]. An intent-to-treat analysis of all participants found that the synchronous technology intervention increased physical activity [44].

#### Asynchronous Technologies

Multimedia Appendix 4 also summarizes the studies related to asynchronous technologies. We found 3 studies that implemented asynchronous technologies [46-48], including an exploratory qualitative study [48], a pre-post study [46], and a fully powered randomized controlled trial [44,47]. Peer support was facilitated through (1) peer-led videos in combination with a website to be used on a tablet by mental health workers to structure discussions about personal recovery [44,46], (2) peer-written emails and peer-led videos on recovery in combination with a noninteractive online psychoeducation program [47], and (3) an interactive website including videos of people with lived experience of mental illness discussing their recovery [46,48]. These interventions aimed at personal recovery [46] and psychiatric self-management [47,48]. Overall, these interventions were feasible, with reports of 80% to 100% engagement [46,47].

Participants who completed the asynchronous technology interventions reported statistically significant benefits in personal recovery [46]. Qualitative findings showed that participants felt “inspired,” “knowing I’m not alone,” and “believing recovery is possible” [48]. One study compared two versions of a peer-supported intervention with a nonpeer-supported psychoeducational text-based website: one consisting of an online psychoeducational program augmented by video testimony and advice from peers, and another consisting of that same program supplemented with email-delivered peer coaching and support [47]. No significant differences were found in any measures of psychiatric symptomatology, anxiety, perceived control over the illness, perceived stigma, functioning, patient satisfaction, or health locus of control [47].

### Community Engagement and Participation

More than half of the studies (16/30, 53%) included community engagement in intervention development [9,22-24,27,31,34-36,38-40,43,46-48]. Four studies used consultative methods of community engagement in intervention development (ie, advice, video content, and information via focus groups) [27,38,47], four studies used active community engagement (ie, co-design as equal partners between scientists and community members) [31,36,46,48], two studies used active and consultative methods [34,35], three studies used a combination of consultative and user-centered designs (ie, focus groups, task analysis, and usability testing) [22-24], two studies used active and user-centered designs (ie, co-design as equal partners between scientists and community members, task analysis, and usability testing) [9], and one study only used a user-centered design [40]. One study did not incorporate any community engagement in intervention development [43]. The remaining studies did not report any community engagement techniques.

Four studies did not report participant engagement in the intervention [25,29,32,40]. In addition, 14 studies were classified as high engagement [9,21,23,24,26,28,31,34-36,38,46,48,49], 7 studies were classified as medium engagement [26,27,30,43-47], and 5 studies were classified as low engagement [20,22,37,39,41]. Among studies that reported high engagement and an intervention development description [23,24,31,34,35,38,47,48], 3 studies included a real-world effectiveness assessment (not efficacy). Of those, studies with the highest level of engagement employed active methods [31,36] or a combination of active and consultative methods [34,35] (see Multimedia Appendix 5).

### Methodological Quality Assessment

Methodological quality was evaluated using an adapted version of the MQRS [16]. MQRS total scores ranged from 2 to 12, with a mean score of 7.5 (SD 2.55) and a median score of 8; six studies had a score10, indicating high methodological quality (see Multimedia Appendix 6). Four studies had a score4, indicating low methodological quality. Many of these studies did not report detailed information about methodology (eg, information about control, follow up, dropout, data analysis). Characteristics associated with methodological quality included use of a manualized intervention design (*k*=9, 69%), provision of sufficient information for replication (*k*=11, 85%), and inclusion of baseline characteristics (*k*=10, 77%).

## Discussion

### Principal Findings

There is growing evidence that digital peer support interventions can improve the lives of people with serious mental illness. This systematic review identified 30 studies that reported on 24 digital peer support interventions. Most of the studies established support for the feasibility, acceptability, and preliminary effectiveness of the interventions with regard to enhancing participants’ functioning, reducing symptoms, and improving program utilization. Peer-delivered and technology-supported interventions demonstrated the most promising evidence for both self-reported biomedical and psychosocial outcomes. Attrition rates varied greatly through all digital peer support platforms. Studies with the highest level of digital health engagement employed active community engagement methods or a combination of active and consultative community engagement methods to develop digital peer support interventions.

The evidence base for digital peer support interventions is predominantly built on single-site trials that included small samples and varying follow-up lengths, which greatly restricted the external validity of these interventions in real-world settings. Digital peer support interventions experience the same issues that are common in the field of digital mental health; thus, well-powered and methodologically rigorous studies are needed to confirm the effect of digital peer support interventions. Billions of dollars are being invested in digital innovation; however, many digital innovations are developed by businesses with profit-making interests, not public health interests. These publicly available digital innovations are marketed without adequate evidence of their effectiveness [50]. Academic and peer support specialists partnering with businesses to rigorously and scientifically appraise digital peer support interventions may lead to the next innovations in peer support as well as digital innovations more broadly.

Peer-delivered and technology-supported interventions demonstrated the most promising evidence for biomedical and psychosocial outcomes. Peer-to-peer networks combined with evidence-based practices predominately included biomedical outcome measures and found positive changes such as reductions in psychiatric symptoms [22,27], maladaptive social cognitions [28], and body weight [25]. In contrast, peer-delivered interventions supported with technology found positive changes in *both* biomedical and psychosocial outcomes such as hope, empowerment, social support, quality of life (no statistically significant improvement) [9,38], recovery [36], medication adherence [35], psychiatric self-management [9], and neurocognitive remediation (statistically significant improvement) [41]. Although the goals of peer support are not typically the same goals as those of traditional clinical services [51], psychosocial outcomes important to service users such as “hope” may act as an important mechanism of health related to biomedical outcomes [52]. Thus, it may be methodologically appropriate to include both biomedical and psychosocial outcomes in order to advance the field of mental health.

Attrition rates varied greatly through all digital peer support platforms. High rates of attrition before achieving intervention effects is a constant challenge in digital psychiatry [53]. Peer support has been noted as a human factor in digital health engagement that facilitates engagement differently than a clinician-patient relationship [10]. For instance, Dr. Fortuna’s model of *reciprocal accountability* indicates that peers promote autonomy, flexible expectations, shared lived experience, and bonding within digital interventions. In contrast, Mohr’s model of *supportive accountability* purports that clinicians foster a therapeutic alliance, positive perceptions of providers’ expertise, and high expectations that the service user has to justify their action or inaction [54]. A prior review found that the addition of technology-mediated peer support might potentially enhance participant engagement and adherence to mental health interventions [13]. However, the varied attrition rates observed in our review suggest more research needs to be done regarding peer support as a human factor in engagement.

Few of the identified studies employed active participation methods such as community-engaged research; rather, the majority of studies employed less involved, consultative methods such as focus groups or requested feedback, or did not report if the community was involved in digital health development. Studies with methodologically appropriate sample sizes to determine effectiveness (ie, outside of a controlled clinical environment or usability testing facilities) that reported the highest level of engagement used active methods such as co-design of the digital programs with peer support specialists as equal partners [31,36] or a combination of active and consultative methods [34,35] (ie, feedback). Applying more participatory research techniques such as community-based participatory research or the Academic-Peer Support Specialists Partnership [11] may facilitate intervention engagement as co-designed interventions become more relevant to the community’s specific needs [8]. Use of co-design and participatory techniques may also improve the generalizability of digital mental health interventions, as many existing programs are often tailored to the needs of individuals who will use them rather than for the broader population of individuals who may benefit [55].

Studies included in this systematic review used a wide range of definitions for peer support. A measurement of fidelity for peer support is in early stages of development [56]; however, to date, a fidelity measure of peer support does not exist, despite a national call for such a measure [57]. As such, it is not known what mechanisms of peer support have a positive or negative impact on biomedical and psychosocial outcomes. Potentially, the variation in peer-delivered interventions supported with technology produces such different results because peer support is currently delivered in widely differing ways. This is potentially in contrast to the other interventions that included asynchronous and synchronous technologies, as the technology was used to guide fidelity. There are multiple models of peer support that are based on different theories, principles, and practices, and they include separate training and statewide Medicaid accreditation procedures. For peer support to become widely recognized as an essential services delivery practice and to ensure quality delivery of peer-based services across diverse settings, a measurement of fidelity is needed.

### Limitations

We acknowledge several limitations of this study. First, we recognize the lack of longitudinal outcomes identified in the included studies, which did not allow us to assess the impact of digital peer services over time. Further research is needed to determine how to sustain improvements in health, especially as people with serious mental illness may need community-based support to augment traditional outpatient clinical support and prevent premature intervention attrition. It would also be important to determine whether the addition of peer services can further contribute to sustained outcomes for people with a lived experience of a serious mental illness using digital interventions. Second, we cannot reliably differentiate which specific aspects of peer support or other health intervention components contributed to positive changes in biomedical and psychosocial outcomes. This highlights an important area of future research focused on examining the specific peer intervention components or peer service–delivery strategies that produce the best outcomes.

### Conclusion

This is the first study to systematically examine digital peer support interventions for people with serious mental illness. It is feasible for peers to use multiple technology modalities to facilitate the delivery of peer support and other evidence-based practices in health care. Similar to other fields in psychiatry, digital health engagement remains an issue. As peer support is an essential recovery service for people with mental health conditions globally [1], this systematic review found that the science of digital peer support is advancing. Advancement of the field requires additional proof-of-concept studies and an examination of digital peer services delivery strategies in combination with high levels of community engagement, as well as further evidence of intervention effectiveness across high, middle, and low-income countries.

## Appendix

Multimedia Appendix 1 PubMed search terms for the systematic review. The search was developed for PubMed and was translated to Embase, Web of Science, Cochrane Central, CINAHL, and PsycInfo.

Multimedia Appendix 2 Current state of the evidence for peer-to-peer networks.

Multimedia Appendix 3 Current state of evidence for peer-delivered interventions supported with technology.

Multimedia Appendix 4 Current state of evidence for asynchronous and synchronous technologies.

Multimedia Appendix 5 Level of community engagement in intervention development and participant engagement rates.

Multimedia Appendix 6 Assessment of methodological quality of included studies.

## Abbreviations

MQRS: Methodological Quality Rating Scale
PICOS: participants, interventions, comparisons, outcomes, and study design
PRISMA: Preferred Reporting Items for Systematic Reviews and Meta-Analyses
