# Supplementary material for: Digital Peer Support Mental Health Interventions for People With a Lived Experience of a Serious Mental Illness: Systematic Review
Source: JMIR Ment Health. 2020 Apr 3;7(4):e16460. doi: 10.2196/16460 (PMC7165313; doi:10.2196/16460)
Supplement: Multimedia Appendix 1 [file mental_v7i4e16460_app1.docx]

(((((peer*[tiab] AND (program*[tiab] OR specialist*[tiab] OR support*

[tiab] OR certifi*[tiab] OR support-specialist*[tiab] OR report*[tiab] OR

decision*[tiab] OR experienc*[tiab] OR intervent*[tiab] OR "social

support"[MESH] OR community-servic*[tiab] AND health*[tiab] OR

coach*[tiab]) OR recovery-coach*[tiab]) OR (share*[tiab] AND (decisionmak*[

tiab] OR "decision making"[MESH] OR support*[tiab] OR

communit*))))) AND ("Schizophrenia Spectrum and Other Psychotic

Disorders"[Mesh] OR "Bipolar and Related Disorders"[mesh] OR "Schizoid

Personality Disorder"[Mesh] OR SMI[tiab] OR Serious-mental-illness*

[tiab] OR serious-mental-disease*[tiab] OR Severe-mental-illness*[tiab]

OR severely-mentally-ill*[tiab] OR persistent-mental-illness*[tiab] OR

chronic-mental-illness*[tiab] OR chronically-mentally-ill*[tiab] OR

schizophren*[tiab] OR schizoaffective*[tiab] OR Schizoid*[tiab] OR

Bipolar-Affective*[tiab] OR Bipolar-disorder*[tiab] OR Bipolar-depression*

[tiab] OR Bipolar-illness*[tiab] OR Paranoia*[tiab] OR Paranoid*[tiab] OR

psychos*[tiab] OR psychot*[tiab] OR mania*[tiab] OR Manic*[tiab] OR

bipola*[tiab])) AND (Youtube[tiab] OR facebook*[tiab] OR smartphone*

[tiab] OR mHealth[tiab] OR eHealth[tiab] OR website*[tiab] OR GPS[tiab]

OR social-media*[tiab] OR app*[tiab] OR telemet*[tiab] OR telemed*

[tiab] OR telepsychiat*[tiab] OR telehealth[tiab] OR telecare*[tiab] OR

telemental-health*[tiab] OR ehealth[tiab] OR mhealth[tiab] OR mobile*

[tiab] OR mobile-health*[tiab] OR mobile-technolog*[tiab] OR mobilephone*[

tiab] OR cellular-phone*[tiab] OR cellphon*[tiab] OR textmessag*[

tiab] OR text-messag*[tiab] OR short-message-service*[tiab]

OR SMS[tiab] OR internet-health*[tiab] OR internet*[tiab] OR online*

[tiab] OR social-media*[tiab] OR tablet*[tiab] OR bluetooth*[tiab] OR

avatar*[tiab] OR GPS[tiab] OR global-positioning-devic*[tiab] OR globalpositioning-

system*[tiab] OR "Geographic Information Systems"[Mesh]

OR geographical-information-system*[tiab] OR "Telecommunications"

[Mesh] OR telecommunic*[tiab] OR virtual-real*[tiab] OR VR[tiab] OR

"Virtual Reality"[Mesh] OR "Virtual Reality Exposure Therapy"[Mesh] OR

website*[tiab] OR web-base*[tiab] OR web-brows*[tiab] OR remoteconsult*[

tiab] OR remote-sens*[tiab] OR artificial-intelligenc*[tiab] OR AI

[tiab] OR game-theor*[tiab] OR user-computer-interfac*[tiab] OR

computer-simulat*[tiab] OR speech-recognition-softwar*[tiab] OR

computer-assisted-therap*[tiab] OR computer-base*[tiab] OR wirelesstechnolog*[

tiab] OR remote-sensing-technolog*[tiab] OR "Medical

Informatics Applications"[Mesh] OR informatics-applic*[tiab] OR

"Telemedicine"[Mesh] OR "Text Messaging"[Mesh] OR "Social Media"

[Mesh] OR "User-Computer Interface"[Mesh] OR "Medical Informatics

Applications"[Mesh] OR "Computer Simulation"[Mesh] OR "Web Browser"

[Mesh] OR "Telemetry"[Mesh] OR "Game Theory"[Mesh] OR

"Smartphone"[Mesh] OR "Computers, Handheld"[Mesh] OR handheldcomputer*[

tiab] OR (computer*[tiab] AND palm-top*[tiab]) OR PDA[tiab]

OR (personal*[tiab] AND digital-assist*[tiab]) OR (tablet*[tiab] AND

computer*[tiab]) OR "Microcomputers"[Mesh] OR personal-comput*[tiab]

OR PC[tiab] OR "Internet"[Mesh] OR "Artificial Intelligence"[Mesh] OR

"Mobile Applications"[Mesh] OR "Online Systems"[Mesh] OR "Cell Phone"

[Mesh] OR "Therapy, Computer-Assisted"[Mesh] OR "Software"[Mesh] OR

"Crowdsourcing"[Mesh] OR "Social Networking"[Mesh] OR "Blogging"

[Mesh] OR "Robotics"[Mesh])
