# Supplementary material for: Digital Peer Support Mental Health Interventions for People With a Lived Experience of a Serious Mental Illness: Systematic Review
Source: JMIR Ment Health. 2020 Apr 3;7(4):e16460. doi: 10.2196/16460 (PMC7165313; doi:10.2196/16460)
Supplement: Multimedia Appendix 2 [file mental_v7i4e16460_app2.docx]

| **Study** | **Country** | **Study Design** | **Study Follow-up** | **Sample Description** | **Intervention** | **Comparison** | **Outcome Measures** | **Results** |
| --- | --- | --- | --- | --- | --- | --- | --- | --- |
| **Moderate Methodological Rigor (Quality Scores 9-12)** | | | | | | | | |
| Kaplan et al [20] | United States | RCT^a^ | 4 and 12 months | N=300, 78% bipolar disorder, 22% schizophrenia-spectrum disorders, 66% female, 87% white, mean age 47 years | (1) Experimental peer support listserv: peer-directed anonymous communication between participants (2) Experimental peer support bulletin board: online bulletin board where participants can post, moderated by peers | Waitlist; participants asked to refrain from using internet peer resources until the end of the study period | Recovery Assessment Scale, Lehman's Quality of Life Interview, The Empowerment Scale, The Medical Outcomes Study Social Support Scale, and questions pertaining to participation and experiences in the online groups | No significant differences were found between groups and outcome measures |
| Kaplan et al [21] | United States | RCT | 3 months | N=60, 87% mood disorder, 13% schizophrenia; 100% female, 89% white, mean age 37 years | Experimental group: online parenting education with weekly 30-minute sessions on child development, stress reduction, mental health and parenting, and positive parenting; supplemented with peer support listserv moderated by provider and mother with lived experience answering questions, providing information, support, and feedback | Control group: access to separate study website containing educational facts about maintaining a healthy lifestyle | Parenting Sense of Competence Scale, Healthy Families Parenting Inventory, Family Coping Inventory, Medical Outcomes Study Social Support Scale | Significant improvement in personal care and role satisfaction in experimental group compared to control group over time; no other significant differences |
| Rotondi et al [22] | United States | RCT | 3 months | N=30, 100% schizophrenia-spectrum disorders; 70% female, 47% white, mean age 38 years N= 21 family/support people; 67% female, 52% white, mean age 52 years | Telehealth intervention: Schizophrenia Online Access to Resources (SOAR), a website containing 3 online therapy groups (for people with schizophrenia only, for family/support people only, multifamily therapy), the ability to ask questions to experts, an archive of previously answered questions, information about activities in the community and news pertaining to mental health issues, and reading materials | Usual care | People with schizophrenia: self-reported stress and social support; whole sample Website Evaluation Instrument | Participants with schizophrenia in the intervention group had significantly less perceived stress, 45% felt some stress while using the website, 69%-81% found the website moderately to extremely easy to use, 44% found the website extremely valuable |
| Rotondi et al [23] | United States | RCT | 3 months | N=30, 100% schizophrenia; 68% female, 46% white, mean age 38 years N=24 family/support people; 62% female, 46% white, mean age 50 years | Telehealth intervention: SOAR, a website containing 3 online therapy groups (for people with schizophrenia only, for family/support people only, multifamily therapy), the ability to ask questions to experts, an archive of previously answered questions, information about activities in the community and news pertaining to mental health issues, and educational reading materials | Usual care | Scale for the Assessment of Positive Symptoms, Knowledge about Schizophrenia Instrument, use of website | Participants with schizophrenia in the intervention group had a significant reduction in positive symptoms, and significant improvement in their knowledge about schizophrenia; family/support people in the intervention group had significant improvement in their knowledge about schizophrenia; people with schizophrenia who had more severe positive symptoms spent more time on the website and visited the website more frequently |
| Schlosser et al. [24] | United States | RCT | 12 weeks | N=43, 100% schizophrenia-spectrum disorders; 38% female, 52% white, mean age 24 years | Personalized Real-time Intervention for Motivational Enhancement (PRIME), a mobile app consisting of a peer community, goal and achievement tracking, and cognitive behavioral-based treatment | Treatment as usual/wait list, after initial 12 weeks participants were given the option of using PRIME | Trust Task, Motivation and Pleasure Self-Report Scale, Role Functioning Scale, Quality of Life Scale, Dysfunctional Attitudes Scale, Beck Depression Inventory, Revised Self-Efficacy Scale, Positive and Negative Syndrome Scale | Participants in the intervention group showed a greater increase in anticipated pleasure, effector to increase likelihood of future social interactions, improvements in depression symptoms and self-efficacy, and decrease in defeatist beliefs |
| **Lower Methodological Rigor (Quality Scores 5-8)** | | | | | | | | |
| Aschbrenner et al [25] | United States | Pre-post | 24 weeks | N=32, 44% major depression, 34% bipolar disorder, 22% schizophrenia-spectrum disorders; 56% female, 97% white, mean age 49 years | PeerFIT included: (1) Weekly 1-hour group weight management sessions facilitated by 2 lifestyle coaches (2) Optional twice weekly 1-hour exercise sessions led by a certified fitness trainer (3) Fitbit and private Facebook group to increase motivation and facilitate self-monitoring and peer support | None | Weight, BMI^b^, 6-Minute Walk Test, Social Provisions Scale | Weight and BMI significantly decreased, weight loss was significantly associated with perceived peer-group support |
| Aschbrenner et al [26] | United States | Pre-post | 24 weeks | N=11, 46% major depression, 27% bipolar disorder, 27% schizophrenia; 73% female, 91% white, mean age 48 years | PeerFIT included: (1) Weekly 90-minute weight management group sessions facilitated by 2 lifestyle coaches and supported by a wellness peer (2) Optional twice weekly 1-hour group exercise sessions led by a certified fitness trainer (3) Fitbit Zip and private Facebook group to increase motivation and facilitate self-monitoring and peer support | None | Program attendance, participant satisfaction, weight, 6-Minute Walk Test | No significant change in weight or fitness; 89% were satisfied with the program, 89% felt the activities were useful, 78% felt the program helped them make progress on health goals |
| Alvarez-Jimenez et al [27] | Australia | Pilot feasibility study | 1 month | N=20, 100% psychotic disorder or mood disorder with psychotic features; 50% female, 45% Anglo Australian, mean age 20 years | HORYZONS online intervention consisting of three components: (1) Peer-to-peer online social networking (2) Individually tailored psychosocial interventions with modules created using evidence-based psychosocial interventions (3) Expert moderation by clinical psychology and vocational workers daily | None | Use of HORYZONS, user experience, safety, Brief Psychiatric Rating Scale, Calgary Depression Scale for Schizophrenia, Beck Anxiety Inventory | 70% felt social networking was useful, 90% felt moderation was supportive, 100% felt HORYZONS was safe and confidential, 60% increased their social connectedness, 55% felt empowered in their recovery process; significant improvements in depression scores |
| Biagianti et al [28] | United States | Pilot feasibility study | 6 weeks | N=27, 89% schizophrenia-spectrum disorders, 11% bipolar disorder with psychosis; 37% female, mean age 28 | Creating Live Interactions to Mitigate Barriers (CLIMB), mobile psychosocial intervention with 2 treatment components: (1) Social cognitive training exercises focusing on gaze perception, visual emotion perception, prosody, theory of mind, affective memory and attribution bias (2) Optimized remote group therapy, master's level clinician led weekly 60-minute group teletherapy sessions, and group text chat between teletherapy sessions | None | Prosody Identification Task, Bell-Lysaker Emotion Recognition Test, Schizophrenia Quality of Life Scale, Positive and Negative Syndrome Scale | Significant improvements in identifications of vocal emotional prosody for happiness and happiness intensity, significant improvements in ability to detect anger |
| Gucci and Marmo [29] | Italy | Quasiexperiment | 6 months | N=446, 100% schizophrenia-spectrum disorder, bipolar disorder, or personality disorder | App2gether, website providing audio/video conference rooms for patients or family and chat with psychologist, psychiatrist, or peer support worker; participants were attending a day-treatment program | (1) Attending day-treatment program (2) Attending transitional day-hospital program (3) No treatment | Global Assessment of Functioning Scale, Quality of Life, Personal and Social Performance, Internalized Stigma Mental Illness Inventory, Empowerment Scale | Participants using app2gether showed a significant decrease in hospital admissions and hospitalization length compared to those not in the IPOLT^c^ program, significant reduction in day-hospital admissions and attendance compared to day-treatment program only and transitional day-hospital program |
| Naslund et al [30] | United States | Exploratory study | 6 months | N=25, 44% major depressive disorder, 35% bipolar disorder, 20% schizophrenia-spectrum disorders; 52% female, 96% white, mean age 49 years | PeerFIT included: (1) Weekly 1-hour group weight management sessions facilitated by 2 lifestyle coaches (2) Optional twice weekly 1-hour exercise sessions led by a certified fitness trainer (3) Fitbit and private Facebook group to increase motivation and facilitate self-monitoring and peer support | None | Facebook interactions, weight, 6-Minute Walk Test, group attendance | Trend towards significant relationship between Facebook interactions and clinically significant weight loss (≥5% weight loss), not significant association between Facebook interactions and improved fitness; 30% of Facebook posts pertained to healthy eating, 19% to program remainders, 18% personal sharing, 13% humor, 8% exercise, 7% community events, and 5% motivation |
| O’Shea et al [31] | United States | RCT | 12 and 18 months | N=131, 100% schizophrenia-spectrum disorder or mood disorder; 100% female, 84% white, mean age 37 years | Three components of the intervention: (1) First 3 months, access to 12 online parenting education sessions focusing on child development, stress reduction, mental illness and parenting, and positive parenting (2) First 12 months, peer support-moderated listserv to answer questions and provide feedback (3) Between months 3 and 6, weekly prerecorded video conversations with experts to reinforce educational material | 12-month access to website containing healthy lifestyle facts | Healthy Families Parenting Inventory subscales: Parent-Child Interaction, Environmental, Mobilizing Resources and Personal Care, and Role Satisfaction; Family Coping Inventory, Parenting Sense of Competence Scale | Intervention effective at decreasing parental stress over time; intervention group significantly greater parental satisfaction at 18 months |
| Simon et al [32] | United States | RCT | 3 weeks | N=118, 100% bipolar disorder, 72% female, 19% minority race/ethnicity | MyRecoveryPlan, an interactive online program designed for people with bipolar disorder consisting of educational, recovery, and self-monitoring modules with peer coaching consisting of group and personalized messages and reminders | MyRecoveryPlan without peer coaching | Use of MyRecoveryPlan | Participants in the coaching group used the following features of MyRecoveryPlan significantly more than the program only group: recovery plan modules daily-self-care and finding support, self-monitoring tools medication and side effects, and discussion groups |
| **Lowest Methodological Rigor (Quality Scores 2-4)** | | | | | | | | |
| O’Leary et al [33] | United States | Pre-post | 2 weeks | N=40 with a self-identified serious mental illness (N=3 bipolar disorder and N=19 depression) or anxiety, autism, eating disorder, or a personality disorder; 70% female, 65% white, mean age 30 years | Guided chat: using Google Docs, chat partners followed a sequence of expressive and reflective prompts based on psychotherapy skills | Unguided chat: participants were instructed to engage in a supportive conversation without prompts | Patient Health Questionnaire, Generalized Anxiety Disorder Scale, participant feedback | Chat type did not have a significant effect on depression and anxiety; anxiety decreased from pre- to post-test regardless of chat type; participants in the guided chat group felt sessions were valuable, powerful, and insightful; participants in the unguided chat group felt the sessions were pleasant and relaxing; both groups disliked the feeling of unwanted responsibility in asking about their chat partner’s problems |
| ^a^RCT: randomized controlled trial.  ^b^BMI: body mass index.  ^c^IPOLT: Institutional Open Light Treatment | | | | | | | | |
