# Supplementary material for: Digital Peer Support Mental Health Interventions for People With a Lived Experience of a Serious Mental Illness: Systematic Review
Source: JMIR Ment Health. 2020 Apr 3;7(4):e16460. doi: 10.2196/16460 (PMC7165313; doi:10.2196/16460)
Supplement: Multimedia Appendix 3 [file mental_v7i4e16460_app3.docx]

| **Study** | **Country** | **Study Design** | **Study Follow-up** | **Sample Description** | **Intervention** | **Comparison** | **Outcome Measures** | **Results** |  |
| --- | --- | --- | --- | --- | --- | --- | --- | --- | --- |
| **Moderate Methodological Rigor (Quality Scores 9-12)** | | | | | | | | | |
| Yamaguchi et al [38] | Japan | RCT^a^ | 6 months | N=53; 70% schizophrenia, 11% depression, 9% bipolar disorder; 42% female; mean age 39 years | Shared decision-making system using Support for Hope and Recovery (SHARE), computer program facilitating treatment decisions with help from peer support specialist | Treatment as usual | Interpersonal Processes of Care Survey Short Form, Patient Activation Measure, Shared Decision Making, Brief Psychiatric Rating Scale, Global Assessment of Functioning, Drug-Induced Extrapyramidal Symptom Scale, Morisky Medication Adherence Scale, Client Satisfaction Questionnaire, Self-Identified Stage of Recovery Parts A and B, World Health Organization Quality of Life | Intervention group significantly higher scores on positive collaboration and positive clinical input subscales, significantly higher positive communication scores, significantly less medication side effects |  |
| **Lower Methodological Rigor (Quality Scores 5-8)** | | | | | | | | | |
| Aschbrenner et al [43] | United States | Mixed methods feasibility pilot study | 6 months | N=10 overweight or obese adults, 60% major depression, 30% schizophrenia-spectrum disorders, 10% bipolar disorder; 90% female, 90% white, mean age 47 years | PeerFIT healthy lifestyle intervention including personalized fitness training, one-on-one and group-based peer health coaching, motivational text messages, and physical activity monitoring via Fitbits or Nike Fuelbands; after 12 weeks, participants transition to peer health coaches | None | Attendance and participant satisfaction, semistructured interviews on participants’ satisfaction, perceived benefits, and recommended modifications | 67% reported the program helped them reach their goals, 67% reported the training materials were very useful, 78% reported it was convenient to participate, 67% would recommend the program, 56% were overall very satisfied with the program |  |
| Finnerty et al [35] | United States | Pre-post | 18 months | N=543; demographics not reported | My Collaborative Health Outcome Information System (MyCHOIS), computer program used to complete a CommonGround shared decision–making report | None | Logs from the computer program containing the following information: creation of user profiles, completion of CommonGround shared decision–making reports | 77% created a user profile, 41% created a CommonGround shared decision–making report, an average of 5 reported completed (excluding those who did not complete a report) |  |
| Finnerty et al [36] | United States | Quasiexperiment | 12 months | N=1416, 40% schizophrenia-spectrum disorders; 56% female, 39% white, mean age 44 | MyCHOIS-CommonGround | Control group matched on baseline demographics and clinical characteristics | Outpatient mental health treatment engagement and antipsychotic medication adherence | MyCHOIS-CommonGround users had significantly higher levels of treatment engagement and were significantly more adherent to medications |  |
| Fortuna et al [9] | United States | Pre-post | 3 months | N=8, 63% major depressive disorder, 25% schizophrenia, 13% bipolar disorder; 88% female, 100% age 60 years or older | PeerTECH eModules on psychoeducation and coping skills training, reviewed weekly in-person with certified peer specialist; smartphone app including access to personalized self-management support, individualized intervention components, medication reminders, chat feature | None | Herth Hope Index, Empowerment Scale, The Medical Outcomes Study Social Support Survey, Illness Management and Recovery Scale, Self-Rated Abilities for Health Practices Scale, Quality of Life and Enjoyment and Satisfaction Questionnaire-Short Form, Self-Efficacy for Managing Chronic Disease Scale | Statistically significant increase in psychiatric self-management skills |  |
| Gulliver et al [44] | Australia | Pre-post | 4 sessions | N=10 individuals with moderate-to-severe mental illness;  N=5 health service staff | Stay Strong app, a structured mental health and substance use intervention on an Apple iPad for collaboration between workers and service users. A peer worker coached participants on use of the app and how to apply concepts. | None | Self-Identified Stages of Recovery, evaluation of Stay Strong app, recovery, and self-efficacy, questions pertaining to acceptability of peer worker | Mode of delivery and program itself deemed not feasible; peer worker seen as highly beneficial |  |
| Salyers et al [37] | United States | Pre-post | 12 and 18 months | N=167, 68% schizophrenia, 43% female, 50% black, mean age not reported | CommonGround was offered on computer kiosks to participants arriving for psychiatric appointments at peer-provided staffed decision-support centers | None | Patient Activation Measure, Autonomy Preference Index, Recovery Assessment Scale, State Hope Scale, assessment of participant involvement and extent to which participant and provided peer worked together as rated by provider | Self-reported symptoms improved overtime, overall recovery attitudes improved, participants who saw the same provider over 18 months improved in provider perceptions of participant involvement |  |
| Sandoval et al [42] | United States | Quasiexperiment | None | N=16; 100% schizophrenia-spectrum disorders; 8% female, 38% white, mean age 24 years | Computer-based neurocognitive training (CBNT), exercises designed to improve alertness, attention, concentration, vigilance, persistence, and processing speed; completed exercises with a peer (another study participant; peer social interaction group) | CBNT without peer | Accuracy in exercises | Peer social interaction significant positive effect on performance in exercise on attention |  |
| **Lowest Methodological Rigor (Quality Scores 0-4)** | | | | | | | | | |
| Korsbek and Tonder [41] | Denmark | Focus group | N/A^b^ | N=78; 100% psychosis or psychosis-like symptoms N=119 providers | Momentum, a smartphone app to support shared decision making through helping participants prepare for treatment appointments, share information with provider, and evaluate appointments. Introduction to Momentum and workshops facilitated by peer supporters | None | Semistructured interview on Momentum's functionality and usability, on supporting consumer’s influence and involvement, its potential to strengthen quality of treatment, motivation for use, and overall experiences | Overall, both consumers and providers felt Momentum was useful in supporting recovery |  |
| Macias et al [39] | United States | Pre-post | 1 month | N=10, 40% schizophrenia-spectrum disorder, 30% bipolar disorder, 30% major depression; 50% female, 100% white, 50% age 50 years or older | WellWave smartphone app designed to support healthy living and psychiatric recovery through: (1) to-do lists with reminders (2) standardized tasks (3) open-ended self-reports (4) suggest from multi-media digital library with articles and motivational videos from peers and mental health professionals | None | Self-rated health quality, perceived health control, stage-of-exercise, accelerometer within smartphone to log date and time of walk, duration in minutes, step count, and speed | Overall improvement in stage-of-exercise, modest improvement in physical health self-ratings |  |
| Mueller et al [40] | United States | Pre-post | 90 days | N=13, 62% major affective disorders, 38% schizophrenia-spectrum disorders; 77% female, 100% white, 46% age 50 years or older | WellWave smartphone app consisting of daily tasks lists, self-report of emotional and physical well-being, text messaging with peer staff | None | Self-rated measures of satisfaction with WellWave, use of Wellwave | 2 participants said they would continue to use WellWave if given the chance, 4 participants enjoyed receiving messages from peer staff, participants suggested adding a feature allowing users to interact with one another |  |
| \| ^a^RCT: randomized controlled trial. \| \| --- \| | | | | | | | | |  |
