# Supplementary material for: Digital Peer Support Mental Health Interventions for People With a Lived Experience of a Serious Mental Illness: Systematic Review
Source: JMIR Ment Health. 2020 Apr 3;7(4):e16460. doi: 10.2196/16460 (PMC7165313; doi:10.2196/16460)
Supplement: Multimedia Appendix 4 [file mental_v7i4e16460_app4.docx]

| **Study** | **Country** | **Study Design** | **Study Follow-up** | **Sample Description** | **Intervention** | **Comparison** | **Outcome Measures** | **Results** |
| --- | --- | --- | --- | --- | --- | --- | --- | --- |
| **Moderate Methodological Rigor (Quality Scores 9-12)** | | | | | | | | |
| Muralidharan et al [45] | United States | RCT^a^ | 3 and 6 months | N=276; 100% SMI^b^; 6% female, 50% black, mean age 54 years | (1) MOVE SMI: in-person weight management program for adults with SMI delivered by mental health provider (8 individual sessions, 16 group sessions) (2) WebMOVE: computerized version of MOVE SMI consisting of 30 online interactive modules (15 on diet, 15 on physical activity) with weekly 25-30 minute phone calls from peer coaches with individualized feedback | Usual care with handout on benefits of weight loss and access to standard weight management program at VA^c^ | International Physical Activity Questionnaire, semi-structured interview to random sample of intervention groups on facilitators and barriers to the program | Significant difference in mean change total MET^d^ minutes from 3 to 6 months in MOVE SMI compared to usual care; significant difference in total MET minutes in WebMOVE compared to usual care; significant increase in moderate and vigorous physical activity in MOVE SMI; significant increase in vigorous physical activity in WebMOVE |
| Proudfoot et al [46] | Australia | RCT | Postintervention 3 and 6 months | N=407; 100% bipolar disorder; 70% female | (1) Bipolar Education Program (BEP): online psychoeducation program consisting of 8 sessions with information pertaining to bipolar disorder, video testimony and advice from peers, and interviews with experts (2) Enhanced condition: BEP plus coaching and support from peers via email | Control group given access to website with brief text on simple facts of bipolar disorder | Brief Illness Perception Questionnaire, Goldberg Anxiety and Depression Scale, Work and Social Adjustment Scale, Rosenberg Self-Esteem Scale, Satisfaction with Life Scale, The Multidimensional Health Locus of Control, additional item on perceived stigma | No significant differences in outcome measures between groups over time |
| Thomas et al [47] | Australia | Pre-post | 3 months | N=10, 100% schizophrenia-spectrum disorders; 10% female, mean age 43 years | Self-Management and Recovery Technology (SMART), an interactive website containing modules on recovery, managing stress, health, oneself, relationships, empowerment, and life, videos of people with lived experience and mental health professionals; participants accessed SMART via tablet during 80 in-person sessions with a mental health worker | None | Questionnaire for the Process of Recovery, Schizophrenia Hope Scale, Friendship Scale, Positive and Negative Syndrome Scale, Depression Anxiety Stress Scale, Assessment of Quality of Life-8 Dimension, Internalized Stigma of Mental Illness Scale, Generalized Self-Efficacy Scale, perceptions of intervention | Significant improvement in measures of recovery, significant decrease in perceived alienation |
| Young et al [48] | United States | RCT | 3 and 6 months | N= 276; 100% SMI, 6% female, 50% black, mean age 54 years | (1) MOVE SMI: in-person weight management program for adults with SMI delivered by mental health provider (8 individual sessions, 16 group sessions) (2) WebMOVE: computerized version of MOVE SMI consisting of 30 online interactive modules (15 on diet, 15 on physical activity) with weekly 25-30 minute phone calls from peer coaches with individualized feedback | Usual care with handout on benefits of weight loss and access to standard weight management program at VA | BMI^d^, WebMOVE completed modules, MOVE SMI session attendance, brief qualitative interview on intervention feasibility and acceptability | WebMOVE group significantly decreased BMI at 6 months, participants in WebMOVE group significantly more likely to lose 5% or more of body weight; WebMOVE group provided positive feedback on modules and peer coaches |
| **Lowest Methodological Rigor (Quality Scores 0-4)** | | | | | | | | |
| Williams et al [49] | Australia | Qualitative interview | 3 months | N=36; 100% SMI; 67% female, mean age 41 years | Use of SMART website for 3 months | None | Semistructured interview with questions exploring overall experience using SMART | Top themes from interviews: “knowing I'm not alone,” “being inspired,” “believing recovery is possible” |
| ^a^RCT: randomized controlled trial.  ^b^SMI: serious mental illness.  ^c^VA: Veterans Administration  ^c^MET: metabolic equivalent of task.  ^d^BMI: body mass index. | | | | | | | | |
