# Supplementary material for: Digital Peer Support Mental Health Interventions for People With a Lived Experience of a Serious Mental Illness: Systematic Review
Source: JMIR Ment Health. 2020 Apr 3;7(4):e16460. doi: 10.2196/16460 (PMC7165313; doi:10.2196/16460)
Supplement: Multimedia Appendix 5 [file mental_v7i4e16460_app5.docx]

|  |  |  |  |  |  |  |  |  |
| --- | --- | --- | --- | --- | --- | --- | --- | --- |
| **Study** | **Engagement** | **Intervention Development Description** | **Consultative Method** | **Active Engagement Method** | **User-Centered Design** | **N/R^a^** | **Engagement Rating^b^** |  |
| **Peer-to-Peer Networks** | | | | | | |  |  |
| Aschbrenner et al [43] | Participants attended an average of 16 weight management sessions, the optional exercise sessions had 28% attendance rate, 100% of participants used the Fitbit and 76% used the private Facebook group | N/R |  |  |  | X | High |  |
| Aschbrenner et al [26] | Attendance rate during first 12 weeks 79% and dropped to 33% during last 12 weeks, an average of 10 optional exercise group sessions were attended | N/R |  |  |  | X | Medium |  |
| Alvarez-Jimenez et al [27] | 95% completed at least 1 full module, 70% used HORYZONS at least 3 out of the 4 weeks | Regular feedback and testing of focus groups with patients and clinicians aided in the development of HORYZONS | X |  |  |  | Medium |  |
| Biagianti et al [28] | 22% completed greater than 16 hours of SCT^c^, 46% of participants completed less than 1 hour a week of training, average attendance rate for ORGT was 85%, average of 1201 group text messages | N/R |  |  |  | X | High |  |
| Kaplan et al [20] | 33% of participants in the bulletin board group never logged in, 76% of participants in both online groups were categorized as low/no participation | N/R |  |  |  | X | Low |  |
| Kaplan et al [21] | In the experimental group 6% discontinued the listserv | N/R |  |  |  | X | High |  |
| Gucci and Marmo [29] | N/R | N/R |  |  |  | X | N/A^d^ |  |
| Naslund et al [30] | 76% of participants agreed to join Facebook group, mean of 4 posts per person, mean of 5 comments per person, mean of 4 likes per person | N/R |  |  |  | X | Medium |  |
| O’Leary et al [33] | Participants completed all chat sessions | N/R |  |  |  | X | High |  |
| O’Shea et al [31] | N/R | N/R |  |  |  | X | N/A |  |
| Rotondi et al [22] | Participants with schizophrenia accessed the website 17,292, family/support people accessed the website 2527; both groups use significantly decreased after the first month | Content of the website was created using information from people with schizophrenia, family members, and professionals (ie, interviews and advisory board) | X |  | X |  | Low |  |
| Rotondi et al [23] | Participants with schizophrenia spent 730 hours on the website; family/support people spent 182 hours on the website | Content of the website was designed specifically for people with schizophrenia, family members, and professionals, and developed through principles of user-centered design | X |  | X |  | High |  |
| Schlosser et al [24] | Participants logged in approximately 4 days a week, average of 23 participant-initiated peer interactions, average of 164 participant-initiated coach interactions, 91% completion rate of challenges | User-center design with people with schizophrenia, family members, treatment providers, and research experts designed the first iteration of PRIME^e^. PRIME was further refined following feedback from participants in a pilot study | X |  | X |  | High |  |
| Simon et al [32] | The inclusion of online peer coaching significantly increased engagement in the program | Peers with bipolar disorder actively contributed to the design of the MyRecoveryPlan website and the peer coaching program |  | X |  |  | High |  |
| **Peer-Delivered Interventions Supported With Technology** | | | | | | |  |  |
| Aschbrenner et al [43] | N/R | N/R |  |  |  | X | N/A |  |
| Finnerty et al [35] | MyCHOIS^f^-CommonGround users had significantly higher levels of treatment engagement and were significantly more adherent to medications. | MyCHOIS-CommonGround refined through feedback from clinics and co-designed with peers | X | X |  |  | High |  |
| Finnerty et al [36] | MyCHOIS-CommonGround users had significantly higher levels of treatment engagement and were significantly more adherent to medications. | MyCHOIS-CommonGround refined through feedback from clinics and co-designed with peers | X | X |  |  | High |  |
| Fortuna et al [9] | 80% participated in 10 or more in-person sessions 74%-88% used the smartphone app weekly, 33%-47% used the smartphone app daily, participants completed 42% of all self-management tasks, 50% reported taking medication daily | User-center design and co-design with people with mental health conditions |  | X | X |  | High |  |
|  |  |  |  |  |  |  |  |  |
| Gulliver et al [44] | Six people with a lived experience of a mental health condition or 100% of the consumer sample used the app in 1 of 4 sessions (note: app usage was only required in 1 of 4 sessions). | Developed by university professors |  |  |  |  | Medium |  |
| Korsbek and Tonder [41] | N/R | Content of Momentum co-created by mental health professionals and consumers |  |  | X |  | N/A |  |
| Macias et al [39] | Participants used WellWave during 94% of the days in the study period, 50% used WellWave every day, 98% response rate to personalized text messages from peer staff, 39% compliance with daily walk prompts | Video content provided by peers and mental health professionals | X |  |  |  | High |  |
| Mueller et al [40] | 2 participants were unable to use WellWave after training, 3 participants used WellWave less than 5 days, 5 participants used WellWave 20 days or more | Video content provided by peers and mental health professionals | X |  |  |  | Low |  |
| Salyers et al [37] | 36% never completed a CommonGround health report, 20% completed 1 report, 14% completed 2 reports, 8% completed 3 reports, 22% completed more than 3 reports | Co-designed with peers |  | X |  |  | High |  |
| Sandoval et al [42] | Comparison group created around the concept of participant no shows | N/R |  |  |  | X | Low |  |
| Yamaguchi et al [38] | The mean number of SHARE uses in the intervention group was 9.23. | N/R |  |  |  | X | Low |  |
| **Asynchronous and Synchronous Technologies** | | | | | | |  |  |
| Muralidharan et al [45] | 18% of MOVE SMI^g^ participants did not attend any session and 0% of the participants attended all the intervention sessions; 24% of WebMOVE participants did not complete any online modules and only 31% of participants completed all the online modules. | N/R |  |  |  | X | Medium |  |
| Proudfoot et al [46] | Significantly more engagement in the enhanced condition compared to BEP only group; engagement was defined as completion of four or more of eight modules. 74% of participants returned four or more modules, 38.8%, returning all eight modules, and 13.3% returning zero workbooks. | Video testimonials and advice provided by peers | X |  |  |  | Medium |  |
| Thomas et al [47] | Participants completed all web sessions | SMART^h^ content was co-designed with people with experience of mental illness |  | X |  |  | High |  |
| Williams et al [49] | All participants attended one interview, 6 agreed to a follow-up interview | SMART content was co-designed with people with experience of mental illness |  | X |  |  | High |  |
| Young et al [48] | 18% of MOVE SMI participants did not attend any session and 0% of participants completed the intervention; 24% of WebMOVE participants did not complete any online modules and 31% of participants completed the intervention. | N/R |  |  |  | X | Medium |  |
| ^a^N/R: not reported.  ^b^Participation rates were categorized as high engagement (75% or more engaged throughout the intervention), medium engagement (74% to 50% engage throughout the intervention), and low engagement (49% or less engage throughout the intervention).  ^c^SCT: Social cognition training  ^d^N/A: not applicable.  ^e^PRIME: Personalized Real-time Intervention for Motivational Enhancement.  ^f^MyCHOIS: My Collaborative Health Outcome Information System.  ^g^SMI: serious mental illness.  ^h^SMART: Self-Management And Recovery Technology. | | | | | | | | |
