# Supplementary material for: Digital Peer Support Mental Health Interventions for People With a Lived Experience of a Serious Mental Illness: Systematic Review
Source: JMIR Ment Health. 2020 Apr 3;7(4):e16460. doi: 10.2196/16460 (PMC7165313; doi:10.2196/16460)
Supplement: Multimedia Appendix 6 [file mental_v7i4e16460_app6.docx]

| **Reference** | **1** | **2** | **3** | **4** | **5** | **6** | **7** | **8** | **9** | **10** | **11** | **12** | **13** | **14** | **15** | **16** | **17** | **18** | **19** | **20** | **21** | **22** | **23** | **24** | **25** | **26** | **27** | **28** | **29** | **30** |
| --- | --- | --- | --- | --- | --- | --- | --- | --- | --- | --- | --- | --- | --- | --- | --- | --- | --- | --- | --- | --- | --- | --- | --- | --- | --- | --- | --- | --- | --- | --- |
| A. Study Design (0-4) | 1 | 0 | 1 | 2 | 2 | 0 | 0 | 0 | 2 | 1 | 1 | 0 | 0 | 1 | 2 | 0 | 0 | 0 | 0 | 0 | 0 | 0 | 0 | 1 | 0 | 1 | 1 | 1 | 0 | 1 |
| B. Replicability (0-1) | 1 | 1 | 1 | 1 | 1 | 0 | 0 | 1 | 1 | 1 | 1 | 1 | 1 | 0 | 1 | 1 | 1 | 1 | 1 | 1 | 1 | 1 | 1 | 1 | 1 | 1 | 1 | 1 | 1 | 1 |
| C. Baseline (0-1) | 0 | 1 | 1 | 1 | 1 | 0 | 1 | 1 | 0 | 0 | 0 | 0 | 1 | 0 | 0 | 1 | 1 | 1 | 1 | 1 | 1 | 1 | 1 | 1 | 0 | 1 | 1 | 0 | 1 | 0 |
| D. Quality Control (0-1) | 1 | 0 | 1 | 1 | 1 | 0 | 0 | 0 | 1 | 1 | 0 | 1 | 1 | 0 | 1 | 0 | 1 | 1 | 1 | 1 | 1 | 1 | 1 | 1 | 1 | 1 | 1 | 1 | 1 | 1 |
| E. Follow-Up Length (0-2) | 0 | 1 | 1 | 1 | 1 | 0 | 0 | 0 | 0 | 2 | 2 | 2 | 1 | 1 | 1 | 0 | 1 | 1 | 0 | 0 | 1 | 0 | 0 | 0 | 0 | 1 | 2 | 0 | 0 | 2 |
| F. Follow-Up Rate (0-2) | 0 | 0 | 2 | 0 | 1 | 2 | 0 | 0 | 2 | 2 | 0 | 0 | 2 | 0 | 1 | 0 | 2 | 2 | 2 | 2 | 2 | 2 | 0 | 2 | 2 | 0 | 2 | 0 | 1 | 0 |
| G. Objective Measurement of Outcomes (0-1) | 1 | 0 | 1 | 1 | 0 | 1 | 1 | 0 | 0 | 0 | 1 | 1 | 1 | 1 | 1 | 1 | 1 | 1 | 0 | 1 | 1 | 1 | 0 | 1 | 1 | 1 | 1 | 0 | 0 | 0 |
| H. Dropouts (0-1) | 0 | 1 | 0 | 0 | 1 | 0 | 0 | 0 | 1 | 1 | 0 | 0 | 0 | 0 | 1 | 0 | 0 | 0 | 0 | 1 | 0 | 1 | 0 | 1 | 1 | 1 | 1 | 0 | 0 | 0 |
| I. Independent (0-1) | 0 | 0 | 0 | 0 | 1 | 0 | 0 | 0 | 0 | 0 | 0 | 0 | 0 | 0 | 0 | 0 | 0 | 0 | 0 | 0 | 0 | 0 | 0 | 0 | 0 | 0 | 0 | 0 | 0 | 0 |
| J. Analyses (0-1) | 1 | 1 | 1 | 1 | 1 | 0 | 0 | 0 | 1 | 1 | 1 | 1 | 1 | 1 | 1 | 1 | 1 | 0 | 1 | 1 | 0 | 1 | 0 | 1 | 1 | 1 | 1 | 1 | 1 | 1 |
| K. Study Site (0-1) | 1 | 1 | 1 | 1 | 0 | 0 | 0 | 0 | 1 | 1 | 1 | 1 | 0 | 1 | 1 | 1 | 0 | 0 | 0 | 0 | 0 | 1 | 1 | 1 | 1 | 1 | 1 | 0 | 0 | 1 |
| L. Collateral (0-1) | 0 | 1 | 1 | 0 | 0 | 0 | 0 | 1 | 0 | 0 | 0 | 0 | 0 | 0 | 0 | 0 | 0 | 0 | 0 | 0 | 0 | 0 | 0 | 0 | 0 | 0 | 0 | 0 | 0 | 0 |
| **Total Quality Score (0 Low to 17 High)** | **6** | **7** | **11** | **9** | **10** | **3** | **2** | **3** | **9** | **10** | **7** | **7** | **8** | **5** | **10** | **5** | **8** | **7** | **6** | **8** | **7** | **9** | **4** | **10** | **8** | **9** | **12** | **4** | **5** | **7** |

*Note*: Study # 1 = Simon et al., 2011; Study # 2 = Salyers et al., 2017; Study # 3 = Yamaguchi et al., 2017; Study # 4 = Young et al., 2017; Study # 5 = Muralidharan et al., 2018; Study # 6 = Macias et al., 2015; Study # 7 = Mueller et al., 2018; Study # 8 = Korsbek & Tenders, 2016; Study # 9 = Kaplan, Solomon, Salzer, & Brusilovskiy, 2014; Study # 10 = Kaplan, Salzer, Solomon, Brusilovskiy, & Cousounis, 2010; Study # 11 = Finnerty et al., 2018a; Study # 12 = Finnerty et al., 2018b; Study # 13 = Aschbrenner, Naslund, Shevenell, Kinney, & Bartels, 2016; Study # 14 = Gucci & Marmo, 2016; Study # 15 = Proudfoot et al., 2012; Study # 16 = Sandoval et al., 2019; Study # 17 = Naslund, Aschbrenner, Marsch, McHugo, & Bartels, 2018; Study # 18 = Ascbrenner, Naslund, Shevenell, Mueser, & Bartels, 2016; Study # 19 = Fortuna et al., 2018; Study # 20 = Alvarez-Jimenez et al., 2013; Study # 21 = Aschbrenner et al., 2015; Study # 22 = Thomas et al., 2016; Study # 23 = Williams, Fossey, Farhall, Foley, & Thomas, 2018; Study # 24 = Schlosser et al., 2018; Study # 25 = Biagianti, Schlosser, Nahum, Woolley, & Vinogradov, 2016; Study # 26 = Rotondi et al., 2005; Study # 27 = Rotondi et al., 2010; Study # 28 = O’Leary, Schueller, Wobbrock, & Pratt, 2018; Study # 29 = Gulliver et al., 2019; Study # 30 = O’Shea et al., 2019
